# Magnetic Cationic Amylose Nanoparticles Used to Deliver Survivin-Small Interfering RNA for Gene Therapy of Hepatocellular Carcinoma In Vitro

**DOI:** 10.3390/nano7050110

**Published:** 2017-05-11

**Authors:** Zhuo Wu, Xiao-Lin Xu, Jun-Zhao Zhang, Xu-Hong Mao, Ming-Wei Xie, Zi-Liang Cheng, Lie-Jing Lu, Xiao-Hui Duan, Li-Ming Zhang, Jun Shen

**Affiliations:** 1 Department of Radiology, Sun Yat-Sen Memorial Hospital, Sun Yat-Sen University, Guangzhou 510120, China; vojoan@hotmail.com (Z.W.); xie_class@126.com (M.-W.X.); czl198601@163.com (Z.-L.C.); luliejingsysu@163.com (L.-J.L.); duanxiaohui-128@163.com (X.-H.D.); 2 Department of Ultrasound, Sun Yat-Sen Memorial Hospital, Sun Yat-Sen University, Guangzhou 510120, China; xlin200398@163.com; 3 Department of Polymer and Materials Science, School of Chemistry, Sun Yat-Sen University, Guangzhou 510275, China; zhjunzh3@mail2.sysu.edu.cn; 4 School of Materials Science and Engineering, Sun Yat-Sen University, Guangzhou 510275, China; m13929581729_1@163.com; 5 Key Laboratory for Polymeric Composite and Functional Materials of Ministry of Education, Guangdong Provincial Key Laboratory for High Performance Polymer-Based Composites, Key Laboratory of Designed Synthesis and Application of Polymer Material, Sun Yat-Sen University, Guangzhou 510275, China

**Keywords:** amylose, small interfering RNA, magnetic resonance imaging, superparamagnetic iron oxide nanoparticles

## Abstract

Amylose is a promising nanocarrier for gene delivery in terms of its good biocompatibility and high transfection efficiency. Small interfering RNA against survivin (survivin-siRNA) can cause tumor apoptosis by silencing a hepatocellular carcinoma (HCC)-specific gene at the messenger RNA level. In this study, we developed a new class of folate-functionalized, superparamagnetic iron oxide (SPIO)-loaded cationic amylose nanoparticles to deliver survivin-siRNA to HCC cells. The cellular uptake of nanocomplexes, cytotoxicity, cell apoptosis, and gene suppression mediated by siRNA-complexed nanoparticles were tested. The results demonstrated that folate-functionalized, SPIO-loaded cationic amylose nanoparticles can mediate a specific and safe cellular uptake of survivin-siRNA with high transfection efficiency, resulting in a robust survivin gene downregulation in HCC cells. The biocompatible complex of cationic amylose could be used as an efficient, rapid, and safe gene delivery vector. Upon SPIO loading, it holds a great promise as a theranostic carrier for gene therapy of HCC.

## 1. Introduction

Hepatocellular carcinoma (HCC) is the most common primary malignant tumor of the liver and one of the most common causes of cancer-related deaths worldwide. Some patients can have curative responses to the treatment of surgery or chemotherapy, whereas those patients with advanced HCC have to be treated with palliative intent [1]. Alternatively, gene therapy has been tested to treat advanced tumors by overcoming the limitations of current therapeutic paradigms. Among them, RNA interference (RNAi), a post-transcriptional gene silencing mechanism, has been emerging as a promising therapeutic strategy [2]. The survivin gene is the smallest member of the inhibitor of apoptosis (IAP) family and plays an important role in oncogenesis, due to its interaction with multiple signaling networks and action in preservation of endothelial cell viability [3]. It has been demonstrated that overexpression of survivin indicates a poor prognosis in up to 70% of HCC patients [4]. In addition, small interfering RNA (siRNA) against survivin has been shown to be able of inducing apoptosis of HCC cells [3]. The success of survivin-targeted RNAi treatment depends mainly on the efficient delivery of specific siRNA to HCC cells, since siRNAs can be rapidly degraded by extracellular RNase and cleared by renal filtration [5]. Therefore, a robust siRNA delivery system is highly desirable for gene therapy of HCC. Moreover, a gene delivery system with image agents, i.e., superparamagnetic iron oxide nanoparticles (SPIO), being loaded would endow the nanoprobe a theranostic nature, which enables noninvasive detection of cancer and simultaneous monitoring of the target therapy. 

Amylose is a natural biopolymer with many advantages, such as abundance, cheapness, biocompatibility, and non-toxicity [6,7]. Its natural linear polysaccharide is composed of glucose residues through α (1 → 4) glycosidic linkages which determines the efficient degradable performance by various enzymes [8]. Amylose can be modified by substituting the hydroxides with quaternary ammonium salt to increase its solubility and enzymatic digestibility in order to meet the requirements of gene delivery. As a nanocarrier backbone, amylose has been constructed into a variety of vehicles for controllable drug and gene delivery, such as flufenamic acid, testosterone, caffeine [9], curcumin, indomethacin [10], image labels [11], therapeutic DNA [12], and RNA [13]. Further modification with a targeted moiety can significantly increase the active and specific accumulation of the nanovehicle in tumor cells of interest. Folate receptors are universally expressed on the surface of tumor cells more frequently than on normal cells [14]. A variety of folate-decorated nanoparticles have been used as promising nanocarriers for solid tumor detection and targeting treatment [15]. Previously, folic acid-conjugated SPIOs were shown to possess good diagnostic sensitivity with magnetic resonance imaging (MRI) in folate-receptor-positive cancer cells [16,17].

In this study, we synthesized a new class of amylose nanoparticles, where the cationic amylose was used as the backbone, folate as the targeting ligand, and SPIO as the image label to deliver survivin-siRNA to HCC cells. The aim of this study was to investigate the potential of folate-functionalized, SPIO-loaded cationic amylose nanoparticles as a new biocompatible polysaccharide-based multifunctional nanoparticle to transfer siRNA against survivin and meet the requirement of simultaneous MRI detection of HCC.

## 2. Materials and Methods

### 2.1. Materials

Amylose (>98%) from potato was purchased from Fluka Co., Ltd. (Buchs, Switzerland). Molar mass distribution of the obtained purified amylose, as detected by gel permeation chromatography (GPC; Waters Corporation, Milford, MA, USA) was: Mn = 83.0 kDa, M_W_ = 107.6 kDa, and a polydispersity index (PDI) = 1.30. siRNA transfection media was purchased from GenePharm Co., Ltd. (Shanghai, China). Glycidyltrimethylammonium chloride (GTA, M_W_ = 151.63 Da) was purchased from Sigma-Aldrich Co. Ltd. (St. Louis, MO, USA). 1-(3-Dimethylaminopropyl)-3-ethylcarbodiimide hydrochloride (EDC·HCl, M_W_ = 191.70 Da), *N*-Hydroxysuccinimide (NHS, M_W_ = 115.09 Da), and folic acid (FA, M_W_ = 441.4 Da) were obtained from Aladdin Reagent Database Inc. (Shanghai, China). 3-(4,5-dimethylthiazol-2-yl)-5-(3-carboxymethoxyphenyl)-2-(4-sulfophenyl)-2H-tetrazolium (MTS) was obtained from Promega Co., Ltd. (Madison, WI, USA). The fluorescent staining agent 4′,6-diamidion-2-phenylindole (DAPI) was purchased from Roche Co., Ltd. (Mannheim, Germany). All other chemicals and reagents were of analytical grade. Survivin siRNA (sense: 5′-CACCGCAUCUCUACAUUCATT-3′; antisense: 5′-UGAAUGUAGAGAUGCGGUGTT-3′) and carboxyfluorescein(FAM)-conjugated scramble RNA (sense: 5′-UUCUCCGAACGUGUCACGUTT-3′; antisense: 5′-ACGUGACACGUUCGGAGAATT-3′) used as negative control were synthesized by GenePharm Co., Ltd. (Shanghai, China).

### 2.2. Synthesis of Magnetic Amylose Nanoparticles

**Figure 1:**
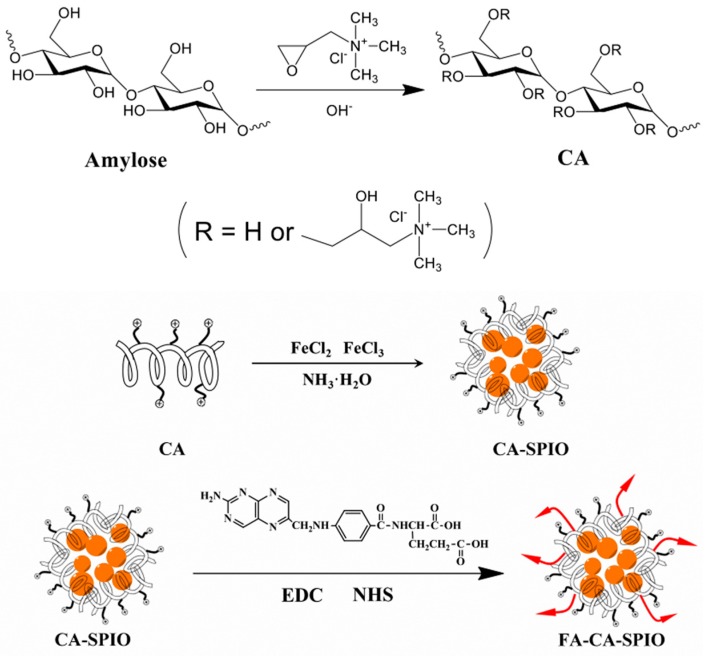
Schematic synthesis of folate-functionalized, superparamagnetic iron oxide nanoparticle-loaded cationic amylose. FA: folate acid; red arrows: FA; CA: cationic amylose; ⊕: positive charges; SPIO: superparamagnetic iron oxide nanoparticle (orange); EDC: 1-(3-dimethylaminopropyl)-3-ethylcarbodiimide; NHS: *N*-hydroxysuccinimide.

Amylose (0.5 g) was dissolved in 25 mL distilled water, and adjusted to pH 12–14 using 4 mol/L NaOH. The solution was mixed and dispersed by ultrasound. Then, 2 g 2,3-epoxypropyl trimethyl ammonium chloride (Sigma-Aldrich Co. Ltd., St. Louis, MO, USA) was slowly added into the solution, and the mixture was stirred at 80 °C for 24 h. After adjusting the pH to 6–7, the mixture was dialyzed for three days (MWCO = 8000–14,000) and lyophilized to obtain the cationic amylase (CA). One-pot synthesis of magnetic nanoparticles was used to obtain SPIO-loaded CA. In brief, 0.2 g CA was dissolved in 20 mL distilled water by continuous stirring. FeCl_3_·6H_2_O (0.2 g) and 0.10 g FeCl_2_·4H_2_O dissolved in 5 mL distilled water was added, stirred, and purged with nitrogen for 30 min. Then, 2.5 mL 25% NH_3_·H_2_O was added under vigorous stirring and the reaction was allowed to proceed for 1 h at 80 °C. The mixture was cooled to room temperature and dialyzed against distilled water for two days (MWCO = 14,000). After centrifugation, the upper aqueous dispersion was collected and lyophilized to obtain SPIO-loaded CA (CA-SPIO). To obtain folate-functionalized CA-SPIO, 20 mg folic acid was dissolved in 4 mL dimethyl sulphoxide (DMSO) by continuous stirring for 1 h, after which 40 mg EDC·HCl and 20 mg NHS were added, the mixture was stirred and allowed to react completely for 4 h away from light to obtain the folic acid ester in DMSO. Then the solution of CA-SPIO (0.1 g CA) was added in a 25 mL flask and acetic acid was added to adjust the pH to 5. After that, the folic acid ester in DMSO was added at room temperature away from light for 48 h. After reaction, the mixture was dialyzed for three days (MWCO = 8000–14,000) and lyophilized to obtain folate-conjugated CA-SPIO (FA-CA-SPIO) (Figure 1).

### 2.3. Synthesis of siRNA-Complexed Magnetic Amylose Nanoparticles

FA-CA-SPIO and CA-SPIO were dissolved in deionized water and stored at 4 °C. Survivin-siRNA was diluted and stored according to the manufacturer’s instructions. To prepare siRNA-complexed magnetic amylose nanoparticles, survivin siRNA and a designated amount of FA-CA-SPIO or CA-SPIO were dissolved separately. The two solutions were mixed by vigorous pipetting, and then the mixture was kept at room temperature for 30 min to allow for the formation of the complexes, survivin-siRNA-complexed FA-CA-SPIO (FA-CA-SPIO/Sur), or survivin-siRNA-complexed CA-SPIO (CA-SPIO/Sur). To prepare negative control RNA-complexed magnetic amylose nanoparticles, FAM-conjugated scramble RNA was added in place of survivin-siRNA in the same manner to obtain FAM-conjugated scramble RNA-complexed CA-SPIO (CA-SPIO/FAM-RNA) or FA-CA-SPIO (FA-CA-SPIO/FAM-RNA). To assess the siRNA condensation ability, gel electrophoresis was performed on a Bio-Rad Sub-Cell^®^ GT electrophoresis cell (Bio-Rad Laboratories, Inc.; Hercules, CA, USA). For the test, nanocomplexes (containing 0.64 μg survivin siRNA) were induced at different *w*/*w* ratios (nanoparticle/siRNA) ranging from 4 to 35, loaded onto 3% agarose gels with EtBr (0.1 µg/mL) and run with Trisacetate-EDTA running buffer at 100 V for 30 min. Visualization and image capture were performed using a UV-transilluminator under a Kodak EDAS 290 digital imaging suite (Fisher Scientific; Pittsburgh, PA, USA). 

### 2.4. Characterization of Nanoparticles

The Fourier transform infrared (FT-IR) spectra was performed on a Nicolet/Nexus 670 FT-IR spectrophotometer (Thermo Nicolet; Madison, WI, USA) at a resolution of 4 cm^−1^ and frequencies ranging from 450 to 4000 cm^−1^ using KBr pellets. ^1^H NMR analysis was carried out on an AVANCE III 400 MHz NMR Spectrometer (BrukerBiospin; Fällanden, Switzerland) at room temperature using DMSO-*d*_6_ or D_2_O as the solvent. The thermogravimetric analyses (TGA) were carried out by using a Pyris 1 thermogravimetric analyzer (Perkin Elmer; Waltham, MA, USA). For TGA, the samples (about 3 mg) were heated from room temperature to 700 °C at a heating rate of 20 °C/min in N2. The X-ray diffraction (XRD) measurements were performed on a SmartLab X-ray diffractometer (Rigaku, Tokyo, Japan) using Cu Kα radiation. The magnetic measurements were carried out with a superconducting quantum interference device (SQUID), model MPMS XL-7 magnetometer (Quantum Design, San Diego, CA, USA). The transmission electron microscopy (TEM) images were obtained on a JEM-2010HR 200kV transmission electron microscope (Jeol Ltd., Tokyo, Japan). For TEM, the sample suspension was prepared by drying a drop (5 μL) of the sample solution on a copper grid coated with amorphous carbon and then blotted with a filter paper after 1 h. After the samples were stained with 10 μL of uranyl acetate solution (2 wt % in water), blotted with a piece of filter paper after 1 min, the grid was finally air dried. The measurements of particle sizes and zeta potentials were performed on a ZetaPALS instrument (Brooken Haven; Long Island, NY, USA). The iron content was detected by using a polarized Zeman atomic absorption spectrometer (AAS; Hitachi Z-200; Tokyo, Japan). For AAS, the samples were dissolved in 1 M HCl solution for thorough release and dissolution of SPIO, then analyzed at the specific Fe absorption wavelength (248.3 nm) based on a pre-established calibration curve. All tests were performed in triplicate.

### 2.5. Cytotoxicity and Apoptosis

For cytotoxic analysis, the influence of FA-CA-SPIO/FAM-RNA and CA-SPIO/FAM-RNA on the viability of HepG2 cells was measured using an MTS assay. HepG2 cells were purchased from the Experimental Animal Center of Sun Yat-Sen University. Briefly, 5 × 10^3^ cells/well were seeded in 96-well plates with 100 μL of complete medium. The nanocomplexes were prepared at various iron concentrations of 0, 5, 10, 20, and 40 μg/mL and at the *w*/*w* ratio of 12. Each nanocomplex concentration was replicated in five wells. Cells in five wells were not transfected and served as controls. FA-CA-SPIO/FAM-RNA or CA-SPIO/FAM-RNA with different concentrations of iron were added and incubated with cells for 30 min. Then, the culture medium was replaced by 100 μL of fresh RPMI-1640 medium. After 48 h of incubation at 37 °C, 10 uL of MTS solution (Promega, Madison, USA) was added to each well. After 3 h of incubation, the absorbance at 492 nm of each well was recorded on a microplate reader (MRXII; Dynex Technologies, Chantilly, VA, USA). For analysis of apoptosis induced by FA-CA-SPIO/Sur or CA-SPIO/Sur, cell apoptosis was measured by flow cytometry. Briefly, 2 × 10^5^ HepG2 cells were seeded in six-well plates in culture medium free of folic acid or supplemented with 1 mmol/L of folic acid. Folic acid was added 30 min before the start of nanoparticle treatment to block folate receptors. After incubation with 24 μL FA-CA-SPIO/Sur or CA-SPIO/Sur for 30 min, cells were washed with phosphate-buffered solution (PBS) three times. Cell apoptosis was assessed by the Annexin V/propidium iodide (PI) double staining method and analyzed using flow cytometry (FACSCalibur, BD Biosciences, San Jose, CA, USA). All experiments were performed in triplicate.

### 2.6. Cellular Uptake Assay

The cellular uptake of nanocomplexes was assessed by Prussian blue staining, fluorescence microscopy, and flow cytometry. HepG2 cells were seeded into 24-well plates at a density of 3 × 10^4^ cells/well and incubated in folic acid-free RPMI-1640 medium or medium supplemented with 1 mmol/L of folic acid for 24 h at 37 °C. Folic acid was added 30 min before the start of nanoparticle treatment to block folate receptors. For Prussian blue staining, 6 μL of FA-CA-SPIO/FAM-RNA or CA-SPIO/FAM-RNA was added to transfect cells at 37 °C for 30 min. After that, the cells were washed with PBS three times and fixed by 1 mL 4% paraformaldehyde solution for 20 min. Then, 1 mL of a 1:1 mixture of 2% potassium ferrocyanate trigydrate and 4% hydrochloric acid (in PBS) was added. The cells were incubated in the dark for 30 min at 37 °C, then washed and counterstained with nuclear fast red for 5 min. Prussian blue staining was examined with an optical microscope (Olympus BX51, Tokyo, Japan). For fluorescence microscopy and flow cytometry assays, HepG2 cells were seeded into the six-well plate at a density of 2 × 10^5^ cells/well and incubated in culture medium free of folic acid or supplemented with 1 mmol/L of folic acid for 24 h. For fluorescence microscopy anlysis, 24 μL of FA-CA-SPIO/FAM-RNA or CA-SPIO/FAM-RNA was added to transfect cells at 37 °C for 30 min, then the DNA-staining agent DAPI (1 mg/mL) was added. Cells were washed three times with PBS, examined by fluorescence microscope (Nikon, Tokyo, Japan). For flow cytometry analysis, 2 mL of PBS containing 24 μL of FA-CA-SPIO/FAM-RNA or CA-SPIO/FAM-RNA was added and incubated at 37 °C for 30 min. Then cells were washed three times with PBS, and analyzed using a flow cytometer (Bio-Rad, Hercules, CA, USA). The experiments were performed in triplicate. The same number of untreated cells were used as controls.

### 2.7. Gene Silencing Effect

To test the gene silencing effect of nanocomplexes, HepG2 cells were seeded in six-well plates at a density of 2 × 10^5^ cells/well. FA-CA-SPIO/Sur, CA-SPIO/Sur and FA-CA-SPIO/Sur plus folic acid were added and incubated at 37 °C for 30 min. Gene silencing of survivin was measured at the mRNA level using the ABI 7500 real-time PCR system. In brief, total RNA was extracted using the RNAiso Plus reagent (TAKARA, Shiga, Japan) according to the manufacturer’s instruction. qPCR was performed according to the protocols from SYBR Premix Ex Taq^™^ kit (TAKARA; Takara Bio, Seta, Japan). The values of sample copies were obtained after quantitative amplification and normalized to glyceraldehyde 3-phosphate dehydrogenase (GAPDH) using the 2^−ΔΔCt^ method. Water was used as negative and quality controls, and each sample was measured in triplicate.

The expression of survivin protein was detected by Western blot analysis. In brief, HepG2 cells were seeded in six-well plates at a density of 2 × 10^5^ cells/well and were incubated with FA-CA-SPIO/Sur, CA-SPIO/Sur, and FA-CA-SPIO/Sur plus folic acid for 30 min. Then the culture medium was replaced by fresh medium and cells were incubated for 48 h. Total protein was extracted using the ProteoJETTM Mammalian Cell Lysis Reagent (Fermentas, Burlington, ON, Canada) with phenylmethanesulfonyl fluoride (PMSF). The protein content was determined using the bicinchoninic acid protein assay kit (Invitrogen, Carlsbad, CA, USA). Ten micrograms of protein were separated on a 10% sodium dodecyl sulfate (SDS) polyacrylamide gel and transferred to polyvinylidene difluoride (PVDF) membranes. The membranes were blocked with 5% fat-free milk in Tris-buffered saline Tween-20 (TBST) for 1 h at room temperature and were subsequently incubated with rabbit anti-human survivin antibody (1:1000 dilution in PBS/Tween; Cell Signaling Technology, Danvers, MA, USA) or rabbit anti-human tubulin antibody (1:10,000; Cell Signaling Technology, Danvers, MA, USA) overnight at 4 °C. Membranes were washed twice with TBST and incubated with HRP-conjugated goat anti-rabbit antibody (Cell Signaling Technology, Danvers, MA, USA) diluted 1:5000 in TBST buffer for 1 h at room temperature. An enhanced chemiluminescence detection reagent (Millipore; Billerica, MA, USA) was then used, and the protein bands were visualized after exposure to X-ray film. Non-transfected cells without transfection were used as controls. The detection of the bands was performed using a VersaDoc model 5000 imaging system quantifying the intensity with Quantity One computer software (Bio-Rad, Hercules, CA, USA). The densitometry of the bands was normalized to the corresponding tubulin band. The experiment was conducted in triplicate.

### 2.8. Magnetic Resonance Imaging

HepG2 cells (1 × 10^4^) were incubated with FA-CA-SPIO or CA-SPIO at various iron concentrations (5, 10, 20, 40 μg/mL) for 30 min, the cells were rinsed with PBS three times, trypsinized, centrifuged, and resuspended in PBS containing 0.5% agarose in 96-well plates for magnetic resonance imaging (MRI). MRI was carried out on a 1.5 T scanner (Intera; Philips Medical Systems, Best, the Netherlands) at room temperature. The sequences included fast field echo T2*-weighted imaging and T2-mapping. T2*-weighted imaging using a fast spin echo sequence with a repetition time (TR)/echo time(TE) = 300/11.5 ms; flip angle = 20°; number of acquisitions (NSA) = 4; acquisition matrix = 268 × 96, field of view (FOV) = 80 mm × 80 mm; and slice thickness/gap = 2/0 mm. T2-mapping was performed by using single-slice multi-echo spin echo sequence for transverse relaxation time (T2 value) calculations with the acquisition parameters: TR/TE = 2600/20–80 ms; four stepped echoes; NSA = 1; acquisition matrix = 308 × 180; FOV = 80 mm × 80 mm; and slice thickness was 2 mm. The experiment was carried out in triplicate.

### 2.9. Statistical Analysis

The data was expressed as the mean ± standard deviation and the average of multiple repeats is shown. The cell viability and apoptosis were analyzed using the Student’s *t*-test. The T2 values were compared using analysis of variance, followed by post-hoc pairwise comparisons using the least significant difference *t*-test. *p* values less than 0.05 was considered to be statistically significant. All statistical tests were performed using the Statistical Package for the Social Sciences software version 17.0 (SPSS Inc., Chicago, IL, USA).

## 3. Results and Discussion

### 3.1. Preparation and Characterization of Cationic Amylose Nanoparticles

The ^1^H NMR spectra of CA and amylose are shown in Figure S1. Compared to amylose, CA showed a new peak at 3.09 ppm, which could be assigned to the –N^+^(CH_3_)_3_ [18]. This indicates that 3-chloro-2-hydroxypropyl trimethylammonium chloride was conjugated with amylose. Figure S2 shows the obvious ultraviolet absorption peak of 280 nm of FA-CA-SPIO compared with CA and CA-SPIO, which suggests the successful conjugation of folic acid to quaternized amylose [19]. Figure S3 shows thermogravimetric curves of SPIO, CA, CA-SPIO, and FA-CA- SPIO, by which the proportion of SPIO could be calculated. Unmodified SPIO has a mass loss of 3.07%, which means no obvious thermolysis of SPIO from 40 °C to 700 °C. The mass loss of CA, CA-SPIO, and FA-CA-SPIO were 87.04%, 60.87%, and 77.97%, respectively, which indicates the part of SPIO in CA-SPIO was 31.17% and SPIO in FA-CA-SPIO was 10.80%. Due to the introduction of SPIO, the thermal degradable component of CA-SPIO and FA-CA-SPIO was decreased, which suggests the successful conjugation of SPIO. 

The iron content of CA-SPIO and FA-CA-SPIO was 900 and 700 μg/mL, respectively. For the resultant CA-SPIO and FA-CA-SPIO, their XRD patterns and magnetization curves were compared to those of primary SPIO, as shown in Figures S4 and S5. The characteristic peaks at 2θ = 30.1°, 35.5°, 43.1°, 53.4°, 57.0°, and 62.6° for SPIO, which were marked respectively by their indices (220), (311), (400), (422), (511), and (440), were also observed for CA-SPIO and FA-CA-SPIO. As shown in Figure S6, the saturation magnetization was found to be 68.7 emu/g for SPIO, 26.8 emu/g for CA-SPIOs, and 24.1 emu/g for FA-CA-SPIO, respectively. The decrease of saturation magnetization of CA-SPIO in comparison with FA-CA-SPIO might be caused by the magnetic dead layer, whereas CA-SPIO or FA-CA-SPIO remained the excellent magnetic responses and were suitable for MR detection.

**Figure 2:**
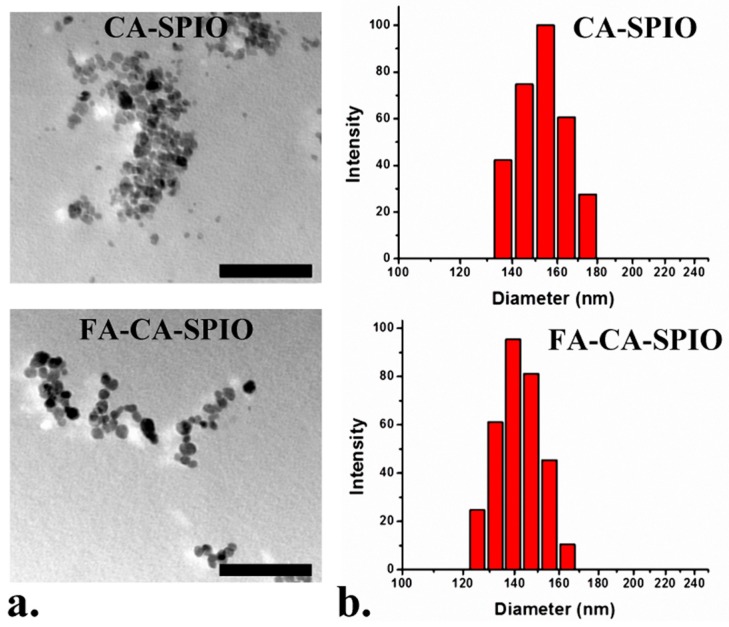
Transmission electron microscope (TEM) images (**a**) and the size distribution of (**b**) CA-SPIO and FA-CA-SPIO. FA, folate acid; CA, cationic amylose; SPIO, superparamagnetic iron oxide nanoparticle. Scale bar: 100 nm.

According to TEM and dynamic light scattering (DLS) measurements, the size of CA-SPIO was 154.4 ± 1.9 nm which was approximately 14 nm larger than the FA-CA-SPIO (139.5 ± 0.8 nm) (Figure 2). Generally, the increase in polymer content would increase the nanoparticle size. In our study, FA-attached nanoparticles were found to have a decreased size. This finding is consistent with previous study on construction of FA-conjugated *N*-trimethyl chitosan nanoparticles, where the mean diameters of fluorescein isothiocyanate-conjugated bovine serum albumin-loaded *N*-trimethyl chitosan (FB-TMC-NP) and FA-conjugated FB-TMC-NP were 184.3 ± 8.3 nm and 176.1 ± 5.0 nm, respectively [20]. They also found that the addition of FA decreased the size of the nanoparticles. In our study, the surface charge of CA-SPIO and FA-CA-SPIO were +8.21 ± 1.42 mV and +7.42 ± 0.34 mV, respectively. In addition, the part of SPIO in CA-SPIO was 31.17% and SPIO in FA-CA-SPIO was 10.80%. The decrease in the zeta potential and iron content might result in the decrease of size of CA-SPIO nanoparticles after the addition of FA. It is known that the small particle size (less than 200 nm) can increase nanoparticle accumulation in tumors because of the enhanced permeability and retention effect [21]. Nanoparticles larger than 200 nm can be rapidly removed by the reticuloendotheliaol system [22]. The size of the cationic amylose nanoparticle is less than that of amylose-based cationic star polymers (average diameter: 234 ± 0.8 nm) [13] and polythyleneimine-polyethylene glycol (PEI-PEG)-based complexes (average diameter: 206 ± 1.9 nm) for siRNA delivery [23]. The relatively small size of the cationic amylose nanoparticles might be more favorable for tumor gene therapy, as they could be more slowly eliminated from blood circulation. Notably, both CA-SPIO and FA-CA-SPIO were positively charged. This cationic nature was critical for nanoparticles to complex with negatively-charged siRNA.

CA-SPIO/Sur and FA-CA-SPIO/Sur were formed at various *w*/*w* ratios ranging from 4, 8, 12, 16, 25, and 35. As shown in Figure S6, with the increase of CA-SPIO or FA-CA-SPIO content in relation to siRNA, the average size of nanocomplexes formed gradually decreased, while the positive potential was gradually increased. The positive charges of cationic amylose nanoparticles can neutralize the negative charges of the siRNA. When the weight ratio reached *w*/*w* = 12, the nanocomplex had a weak positive charge and a size of approximately 150 nm. This indicates that cationic amyloses can effectively condense siRNA to form stable nanoparticles. In the following experiments, the weight ratio was kept at *w*/*w* = 12 to obtain various siRNA-complexed nanoparticles. The complexing of siRNA to CA-SPIO was further examined by electrophoretic mobility on agarose gels. As shown in Figure S7, the migration of siRNA was completely retarded when the *w*/*w* ratio of FA-CA-SPIO and CA-SPIO to siRNA was equal to, or greater than, 12. Moreover, no significant difference was found in the siRNA condensing ability between FA-CA-SPIO/Sur and CA-SPIO/Sur, which suggests that folate functionalization of nanoparticles did not influence their ability to condense siRNA.

### 3.2. Cellular Uptake

**Figure 3:**
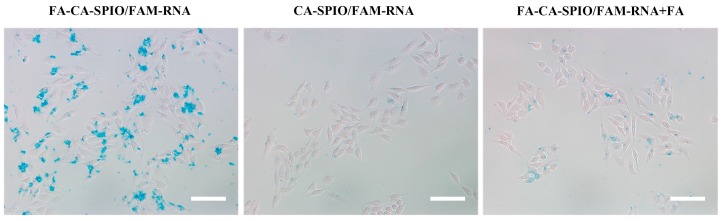
Cell uptake of nanoparticles. Representative Prussian blue staining micrographs show obvious uptake of iron nanoparticles in HepG2 cells transfected with the folate-conjugated superparamagnetic iron oxide nanoparticles (SPIO)-loaded cationic amylose complexed with FAM-conjugated scramble siRNA (FA-CA-SPIO/FAM-RNA), compared with those transfected with CA-SPIO/FAM-RNA without folic acid modification (CA-SPIO/FAM-RNA) and FA-CA-SPIO/FAM-RNA plus FA. Scale bar: 100 nm.

**Figure 4:**
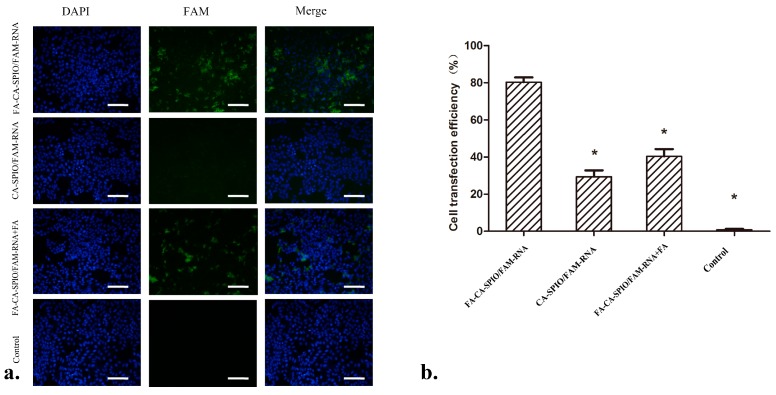
In vitro cell transfection efficiency analysis. (**a**) Representative fluorescence microscopy micrographs show the uptake of siRNA in HepG2 cells after 30 min incubation with the superparamagnetic iron oxide nanoparticles (SPIO)-loaded cationic amylose complexed with FAM-labeled scramble siRNA (CA-SPIO/FAM-RNA), folate-conjugated CA-SPIO/FAM-RNA (FA-CA-SPIO/FAM-RNA), FA-CA-SPIO/FAM-RNA plus FA and untreated cells (control). Scale bar: 100 μm; and (**b**) flow cytometric data of cells incubated with FA-CA-SPIO/FAM-RNA, CA-SPIO/FAM-RNA, FA-CA-SPIO/ FAM- RNA plus FA and untreated cells (control). *: *p* < 0.05 compared with FA-CA-SPIO/FAM-RNA group.

Prussian blue staining showed that cells treated with FA-CA-SPIO/Sur had more stained particles than those treated with CA-SPIO/Sur or FA-CA-SPIO/Sur plus FA (Figure 3). These results confirm the specific uptake of nanocomplexes mediated by FA. It is noted that only 30 min incubation time was needed for siRNA transfection, which is much shorter than that of 4–48 h reported for using other polymeric vectors in order to transfer siRNA into cells [13,23]. This suggests a high transfection efficiency of the SPIO-loaded cationic amylose nanoparticles. Furthermore, HepG2 cells treated with FA-CA-SPIO/FAM-RNA showed significantly stronger FAM green fluorescent signals in comparison with cells treated with CA-SPIO/FAM-RNA or FA-CA-SPIO/FAM-RNA blocked with FA. These results indicate that FA-modified CA-SPIO can effectively deliver siRNA into HepG2 cells via receptor-mediated active targeting pathway. As shown in Figure 4, the percentage of FAM-positive cells incubated with FA-CA-SPIO/FAM-RNA, CA-SPIO/FAM-RNA and FA-CA-SPIO/FAM-RNA plus FA were 80.3 ± 4.5%, 29.3 ± 6.0%, and 40.3 ± 6.8%, respectively. The percentage of positive cells in FA-CA-SPIO/FAM-RNA transfection was significantly higher than that transfected with CA-SPIO/FAM-RNA or FA-CA-SPIO/FAM-RNA plus FA. This finding is consistent with a previous study, where FA as a surface-conjugated molecule was demonstrated to significantly increase the targeting properties of amylose nanoparticles [24]. This might be explained by receptor-mediated endocytosis being much more efficient than nonspecific adsorptive endocytosis in transporting macromolecules into cells.

### 3.3. Cytotoxicity and Apoptosis

**Figure 5:**
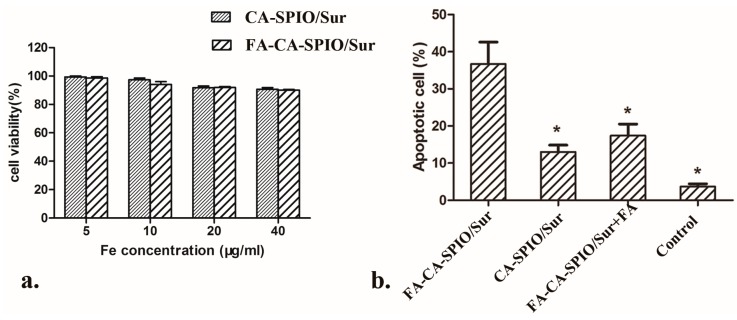
The cytotoxicity and apoptosis of cells transfected with various nanocomplexes. (**a**) A graph showing the viability of HepG2 cells incubated with the superparamagnetic iron oxide nanoparticle (SPIO)-loaded cationic amylose complexed with FAM-labeled scramble siRNA (CA-SPIO/FAM-RNA), and folate-conjugated CA-SPIO/FAM-RNA (FA-CA-SPIO/FAM-RNA); and (**b**) a graph showing the apoptotic rates of HepG2 cells treated with CA-SPIO/Sur, FA-CA-SPIO/Sur, FA-CA-SPIO/Sur plus FA, and untreated cells (control). *: *p* < 0.05 compared with FA-CA-SPIO/Sur group.

The MTS assay was used to assess the cell viability. As shown in Figure 5, when the iron concentration of nanocomplexes increased from 5 to 40 μg/mL, the viability of cells incubated with CA-SPIO/FAM-RNA or FA-CA-SPIO/FAM-RNA remained above 90%, though a slight decrease of cell viability was observed. Meanwhile, the MTS assay did not reveal significant differences in the cytotoxicity between HepG2 cells treated with FA-CA-SPIO/FAM-RNA and CA-SPIO/FAM-RNA. These results demonstrate that FA-CA-SPIO and CA-SPIO have high biocompatibility and are good candidates for gene delivery vectors. The apoptotic cells were quantified by flow cytometry. The mean apoptotic rates for cells treated with FA-CA-SPIO/Sur, CA-SPIO/Sur, FA-CA-SPIO/Sur plus FA, and untreated cells were 36.7 ± 10.3%, 13.3 ± 3.2%, 17.4 ± 5.4%, and 3.2 ± 1.8%, respectively. The apoptotic rate in the FA-CA-SPIO/Sur group was significantly higher than the other three groups.

### 3.4. Survivin Gene Suppression

**Figure 6:**
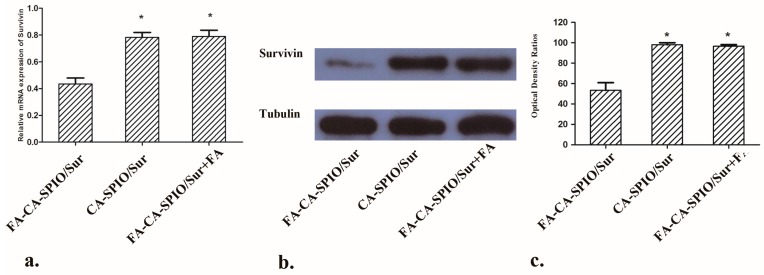
Survivin gene expression analysis. (**a**) A graph showing the quantification of survivin mRNA expression in cells treated with the superparamagnetic iron oxide nanoparticle (SPIO)-loaded cationic amylose complexed with survivin siRNA (CA-SPIO/Sur), folate-conjugated CA-SPIO/Sur (FA-CA-SPIO/Sur) and FA-CA-SPIO/Sur plus FA. *: *p* < 0.05 FA-CA-SPIO/Sur group; (**b**) Western blot analysis show protein expression level of survivin in HepG2 cells incubated with various nanocomplexes of CA-SPIO/Sur, FA-CA-SPIO/Sur and FA-CA-SPIO/Sur plus FA. Tubulin is used as a loading control; and (**c**) a graph showing the corresponding optical density ratios of survivin to tubulin bands as detected by Western blot analysis in CA-SPIO/Sur, FA-CA-SPIO/Sur, and FA-CA-SPIO/Sur plus FA groups. *: *p* < 0.05 compared with FA-CA-SPIO/Sur group.

The ability of nanocomplexes to downregulate the level of survivin mRNA and protein expression in HepG2 cells was evaluated by RT-PCR and Western blot analysis. The relative mRNA expression of the cells incubated with various nanocomplexes is shown in Figure 6a. Compared to the mRNA level of untreated cells that was taken as 1.0, FA-CA-SPIO/Sur transfection reduced the mRNA levels of survivin in HepG2 cells to 0.43 ± 0.07, CA-SPIO/Sur reduced mRNA levels to 0.78 ± 0.06, and FA-CA-SPIO/Sur plus reduced mRNA levels to 0.79 ± 0.08, respectively. The delivery of siRNA via FA-CA-SPIO caused significant decrease of the mRNA expression of survivin. The gene silencing effect of FA-CA-SPIO/Sur is close to another amylose-based cationic star polymers (34% of control mRNA level) [13]. The survivin gene suppression effect was further assessed by Western blot analysis. As shown in Figure 6b, the level of survivin protein expression in HepG2 cells treated with FA-CA-SPIO/Sur was decreased in comparison to cells incubated with CA-SPIO/Sur or FA-CA-SPIO/Sur plus FA. Collectively, cationic amylose with folate functionalization can significantly enhance the endocytosis of siRNA to downregulate the survivin gene expression in HepG2 cells. Amylose nanoparticles can be used as a desirable carrier for siRNA delivery. It should be noted that our study was conducted in cell culture systems in vitro. For In vivo application of this gene delivery carrier, many challenges, including short circulation time, non-targeted distribution, inefficient endosomal escape, and the balances between the transfection efficiency, targeting specificity, particle size, biocompatibility, and the cytotoxicity in the environment should be overcome [25,26]. Moreover, the removal of gene delivery vectors by the reticulo-endothelial system may also decrease the efficacy of therapeutic genes of interest [26]. Furthermore, the positive results of gene delivery vectors obtained from small animals should be transferred to larger animals and humans, since gene therapeutics have not yet been fully developed and tested in clinical trials [27].

### 3.5. Magnetic Resonance Imaging (MRI)

**Figure 7:**
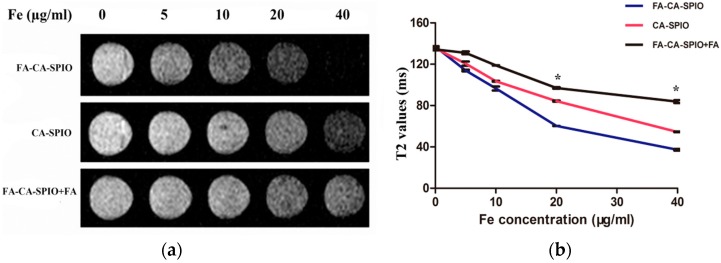
MRI of the cell uptake. (**a**) T2*-weighted image shows the signal intensity of HepG2 cells incubated with superparamagnetic iron oxide nanoparticle (SPIO)-loaded cationic amylose (CA-SPIO), folate-functionalized CA-SPIO, and FA-CA-SPIO plus FA; and (**b**) a graph showing the T2 values of the cells incubated with various nanocomplexes at different iron concentration. *: *p* < 0.05.

The MRI visibility of amylose nanoparticles was assessed by observing T2*-weighted signal intensity and by measuring T2 value of HepG2 cells when incubated with different doses of nanoparticles on a clinical 1.5 T scanner. As shown in Figure 7, the signal intensity of cells showed a gradual decrease with the increasing of iron concentrations of nanoparticles from 5 to 40 µg/mL. The T2 values measured from T2-maps were used to quantitatively evaluate the uptake efficiency of nanoparticle. At 20 μg/mL iron concentration or above, the T2 value of HepG2 cells incubated with FA-CA-SPIO was significantly decreased in comparison to cells treated with CA-SPIO. Such an effect on the signal intensity was inhibited by folic acid blocking. These results suggest that FA-CA-SPIO can be efficiently transferred to HepG2 cells with the help of FA-mediated targeting ability. As such, it enables an MRI-visible gene delivery for tumor cells.

## 4. Conclusions

In conclusion, our study results demonstrated that cationic amylose nanoparticles can act as effective carriers for siRNA delivery and showed a high transfection efficiency, thereby exerting a silencing effect of the survivin gene in HCC cells. With the functionalization with SPIO and FA, this new class of nanovehicle not only has a capability of targeted gene delivery to cancer cells, but also makes the delivery process detectable by MRI, which holds great promise in theranostic gene therapy of HCC in the future. However, the real promise of this class of nanocarriers will only be realized by studies in animal models and, ultimately, in clinical trials, which will be further investigated in our future study.

## Supplementary Materials

The following are available online at http://www.mdpi.com/2079-4991/7/5/110/s1. Figure S1: ^1^H NMR spectra of amylose and cationic amylose (CA), Figure S2: UV-VIS spectra of cationic amylose (CA), superparamagnetic iron oxide nanoparticle-loaded CA(CA-SPIO) and folate-functionalized CA-SPIO (FA-CA-SPIO), Figure S3: Thermogravimetric (TG) curves of SPIO, CA, CA-SPIO and FA-CA-SPIO, Figure S4: X-ray diffraction diagrams of SPIO, CA, CA-SPIO, and FA-CA-SPIO, Figure S5: Magnetization curve of SPIO, CA, CA-SPIO, and FA-CA-SPIO, Figure S6: The particle size and zeta potentials of CA-SPIO and FA-CA-SPIO complexed with survivin siRNA formed at various *w*/*w* ratios, Figure S7: Gel retardation assay of CA-SPIO and FA-CA-SPIO complexed with survivin siRNA.
