# Supplementary material for: Magnetic Cationic Amylose Nanoparticles Used to Deliver Survivin-Small Interfering RNA for Gene Therapy of Hepatocellular Carcinoma In Vitro
Source: Nanomaterials (Basel). 2017 May 11;7(5):110. doi: 10.3390/nano7050110 (PMC5449991; doi:10.3390/nano7050110)
Supplement: Supplementary file 1 [file nanomaterials-07-00110-s001.pdf]

Magnetic Cationic Amylose Nanoparticles Used to Deliver Survivin-Small Interfering RNA for Gene Therapy of Hepatocellular Carcinoma In Vitro

Zhuo Wu [1,†], Xiao-Lin Xu [2,†], Jun-Zhao Zhang [3,†], Xu-Hong Mao [4], Ming-Wei Xie [1], Zi-Liang Cheng [1], Lie-Jing Lu [1], Xiao-Hui Duan [1], Li-Ming Zhang [3,4,5,*] and Jun Shen [1,*]

[1] Department of Radiology, Sun Yat-Sen Memorial Hospital, Sun Yat-Sen University, Guangzhou 510120, China; vojoan@hotmail.com (Z.W.); xie_class@126.com (M.-W.X.); czl198601@163.com (Z.-L.C.); luliejingsysu@163.com (L.-J.L.); duanxiaohui-128@163.com (X.-H.D.)

[2] Department of Ultrasound, Sun Yat-Sen Memorial Hospital, Sun Yat-Sen University, Guangzhou 510120, China; xlin200398@163.com

[3] Department of Polymer and Materials Science, School of Chemistry, Sun Yat-sen University, Guangzhou 510275, China; zhjunzh3@mail2.sysu.edu.cn

[4] School of Materials Science and Engineering, Sun Yat-sen University, Guangzhou 510275, China; m13929581729_1@163.com

[5] Key Laboratory for Polymeric Composite and Functional Materials of Ministry of Education, Guangdong Provincial Key Laboratory for High Performance Polymer-based Composites, Key Laboratory of Designed Synthesis and Application of Polymer Material, Sun Yat-sen University, Guangzhou 510275, China

* Correspondence: ceszhlm@mail.sysu.edu.cn (L.-M.Z.); shenjun@mail.sysu.edu.cn (J.S.); Tel.: +86-20-8411-2354 (L.-M.Z.); Tel./Fax: +86-20-8133-2702 (J.S.)

† These authors contribute equally to this work.

Supplementary Materials

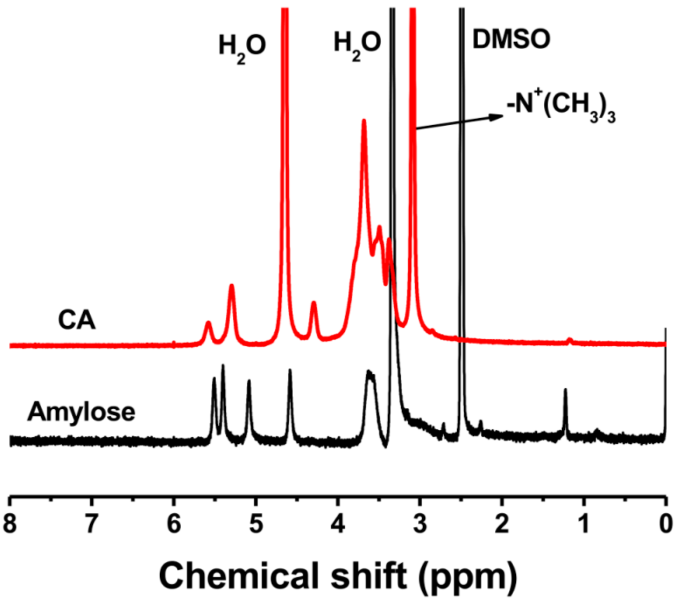

Figure S1. ^{1}H NMR spectra of amylose and cationic amylose (CA). DMSO:dimethyl sulphoxide. CA showed new peak at 3.09 ppm compared with amylose, which could be assigned to the $-N^+(CH_3)_3$.

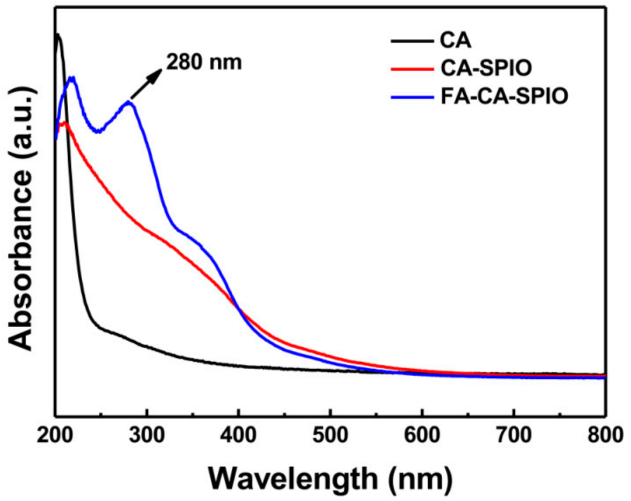

Figure S2. Ultra violet (UV)-vis spectra of cationic amylose (CA), superparamagnetic iron oxide nanoparticle-loaded CA(CA-SPIO) and folate-functionalized CA-SPIO (FA-CA-SPIO). Obvious ultraviolet absorption peak of 280 nm of FA-CA-SPIO compared with CA and CA-SPIO suggests the successful conjugation of folic acid to the quaternized amylose.

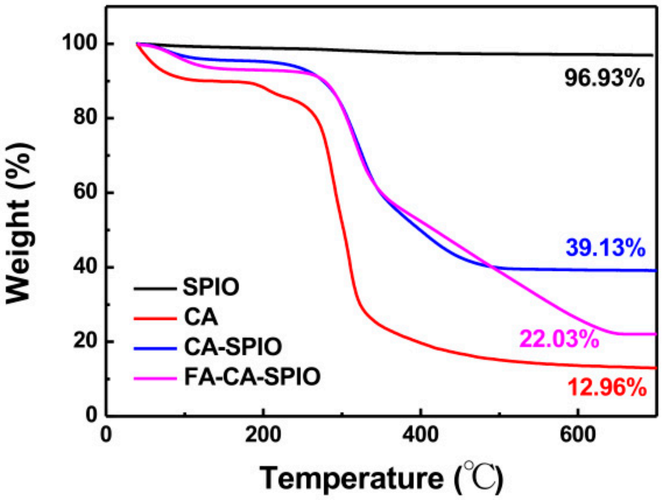

Figure S3. Thermogravimetric (TG) curves of superparamagnetic iron oxide nanoparticles (SPIO), cationic amylose (CA), SPIO-loaded CA(CA-SPIO) and folate-functionalized CA-SPIO (FA-CA-SPIO). Unmodified SPIO has a mass loss of 3.07%. The mass loss of CA, CA-SPIO and CA-FA-SPIO were respectively 87.04 %, 60.87 % and 77.97 %, which indicates the part of SPIO in CA-SPIO was 31.17% and SPIO in CA-FA-SPIO was 10.80%.

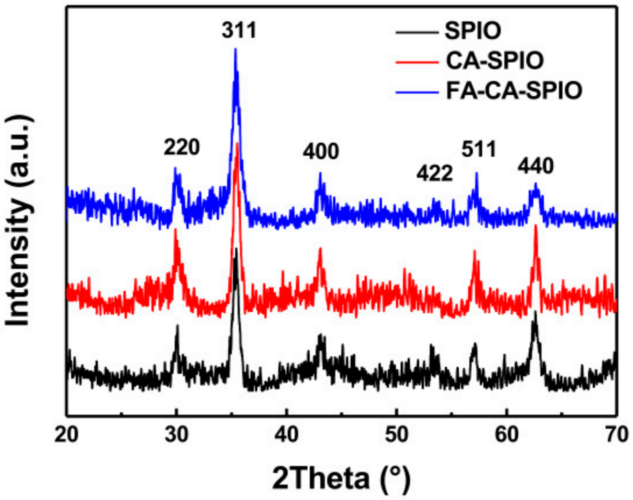

Figure S4. X-ray diffraction diagrams of superparamagnetic iron oxide nanoparticles (SPIO), SPIO-loaded cationic amylose (CA-SPIO) and folate-functionalized CA-SPIO (FA-CA-SPIO). The characteristic peaks at 2θ= 30.1°, 35.5°, 43.1°, 53.4°, 57.0° and 62.6° for SPIO, which were marked respectively by their indices (220), (311), (400), (422), (511) and (440), were also observed for CA-SPIO and FA-CA-SPIO.

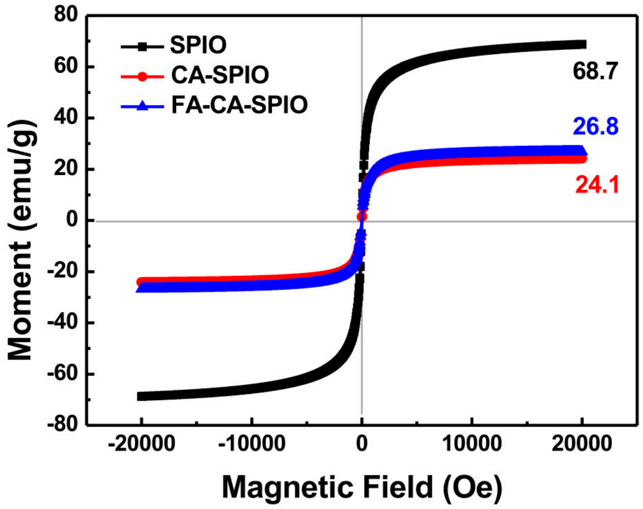

Figure S5. Magnetization curve of superparamagnetic iron oxide nanoparticles (SPIO), SPIO-loaded cationic amylose (CA-SPIO) and folate-functionalized CA-SPIO (FA-CA-SPIO). The saturation magnetization was 68.7 emu/g for SPIO, 26.8 emu/g for CA-SPIOs and 24.1 emu/g for FA-CA-SPIO. CA-SPIO or CA-FA-SPIO remained the excellent magnetic responses.

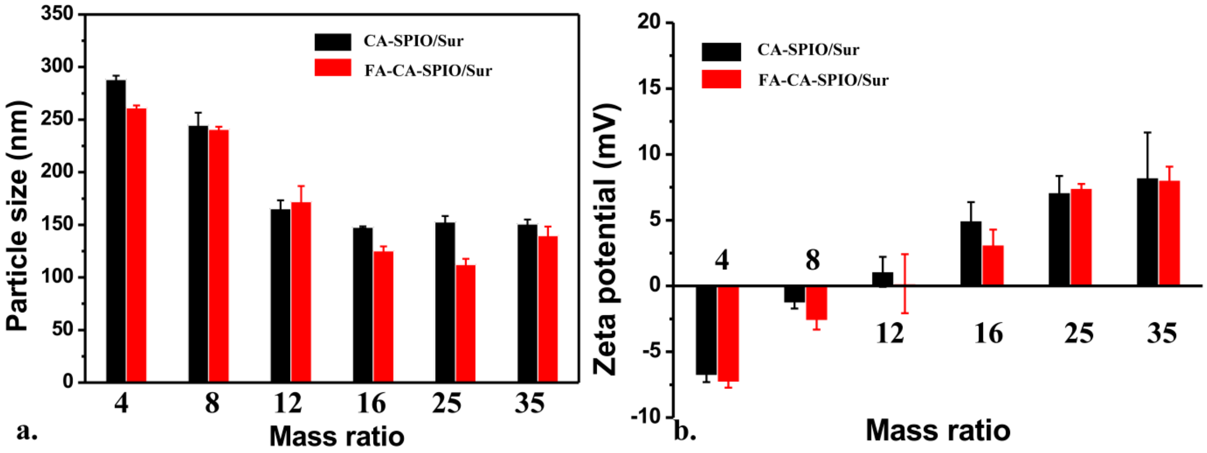

Figure S6. The particle size **(a)** and zeta potentials **(b)** of the superparamagnetic iron oxide nanoparticles (SPIO)-loaded cationic amylose (CA-SPIO) and folate-functionalized CA-SPIO (FA-CA-SPIO) complexed with survivin siRNA (Sur) formed at various w/w ratios. With the increase of CA-SPIO or CA-FA-SPIO content in relation to siRNA, the average size of nanocomplexes gradually decreased, while the positive potential was gradually increased. When the weight ratio reached w/w = 12, the nanocomplex had a weak positive charge and a size of approximately 150 nm.

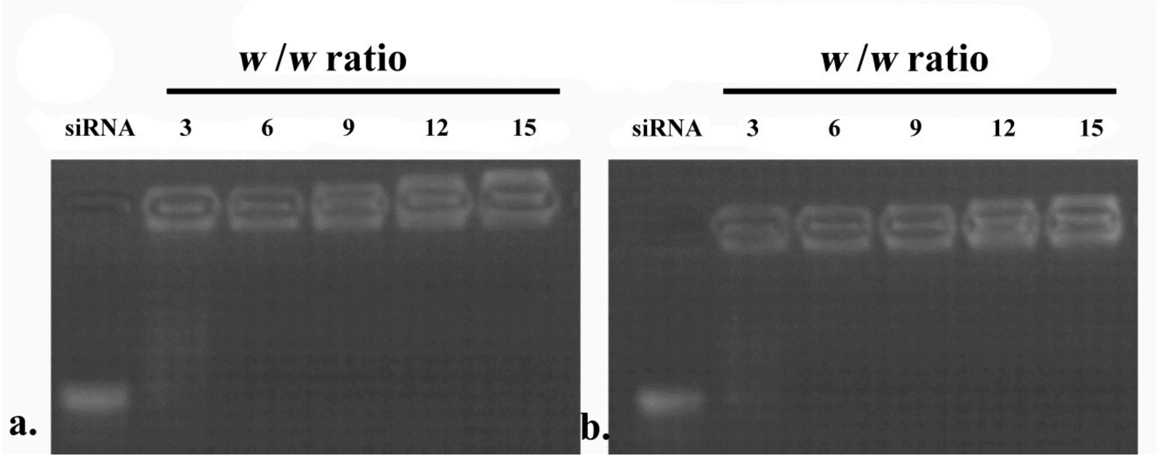

Figure S7. Gel retardation assay of superparamagnetic iron oxide nanoparticles (SPIO)-loaded cationic amylose (CA-SPIO) **(a)** and folate-functionalized CA-SPIO (FA-CA-SPIO) complexed with survivin siRNA (Sur)**(b)**. Amylose nanoparticles were formed at various w/w ratios from 3 to 15. siRNA bands dissociated from nanoparticles were separated by electrophoresis and visualized by an ultra violet (UV) imaging system. Complete siRNA condensation was formed at w/w ratio of 12. Lane 2-7: nanoparticles formed at w/w ratios of 3, 6, 9, 12, and 15; Lane 1: naked siRNA as a control.
